# Comparison of risk prediction scoring systems for ward patients: a retrospective nested case-control study

**DOI:** 10.1186/cc13947

**Published:** 2014-06-26

**Authors:** Shun Yu, Sharon Leung, Moonseong Heo, Graciela J Soto, Ronak T Shah, Sampath Gunda, Michelle Ng Gong

**Affiliations:** 1 Division of Critical Care Medicine, Department of Medicine, Montefiore Medical Center, 111 East 210th Street, Bronx, NY 10467, USA; 2 Department of Epidemiology and Population Health, Albert Einstein College of Medicine, 1300 Morris Park Avenue, Bronx, NY 10461, USA; 3 Department of Medicine, St. Barnabas Hospital 4422 3rd Avenue, New York, NY 10457, USA

## Abstract

**Introduction:**

The rising prevalence of rapid response teams has led to a demand for risk-stratification tools that can estimate a ward patient’s risk of clinical deterioration and subsequent need for intensive care unit (ICU) admission. Finding such a risk-stratification tool is crucial for maximizing the utility of rapid response teams. This study compares the ability of nine risk prediction scores in detecting clinical deterioration among non-ICU ward patients. We also measured each score serially to characterize how these scores changed with time.

**Methods:**

In a retrospective nested case-control study, we calculated nine well-validated prediction scores for 328 cases and 328 matched controls. Our cohort included non-ICU ward patients admitted to the hospital with a diagnosis of infection, and cases were patients in this cohort who experienced clinical deterioration, defined as requiring a critical care consult, ICU admission, or death. We then compared each prediction score’s ability, over the course of 72 hours, to discriminate between cases and controls.

**Results:**

At 0 to 12 hours before clinical deterioration, seven of the nine scores performed with acceptable discrimination: Sequential Organ Failure Assessment (SOFA) score area under the curve of 0.78, Predisposition/Infection/Response/Organ Dysfunction Score of 0.76, VitalPac Early Warning Score of 0.75, Simple Clinical Score of 0.74, Mortality in Emergency Department Sepsis of 0.74, Modified Early Warning Score of 0.73, Simplified Acute Physiology Score II of 0.73, Acute Physiology and Chronic Health Evaluation II of 0.72, and Rapid Emergency Medicine Score of 0.67. By measuring scores over time, it was found that average SOFA scores of cases increased as early as 24 to 48 hours prior to deterioration (*P* = 0.01). Finally, a clinical prediction rule which also accounted for the change in SOFA score was constructed and found to perform with a sensitivity of 75% and a specificity of 72%, and this performance is better than that of any SOFA scoring model based on a single set of physiologic variables.

**Conclusions:**

ICU- and emergency room-based prediction scores can also be used to prognosticate risk of clinical deterioration for non-ICU ward patients. In addition, scoring models that take advantage of a score’s change over time may have increased prognostic value over models that use only a single set of physiologic measurements.

## Introduction

Risk prediction scores provide an important tool for clinicians by allowing standardized and objective estimations of mortality for both research and clinical decision-making purposes. Many such scoring systems have been successfully developed in recent decades for emergency room (ER) and intensive care unit (ICU) patients [1-9]. In subsequent validation studies, many of these scores have been confirmed to be clinically useful tools in the ER [10-15] and ICU [16-23] with good discriminatory power.

Following the increasing prevalence of rapid response teams (RRTs) is a demand for an accurate risk-stratification tool for patients on the wards who are at-risk for clinical deterioration and subsequent ICU admission. A number of “track and trigger” systems have been developed for this purpose, designed to trigger reassessment by the medical team whenever tracked physiologic parameters reached an arbitrary critical level. However, validation of these track-and-trigger systems has generally revealed poor sensitivity, poor positive predictive value, and low reproducibility [24-27]. Although prediction scores based on ER and ICU data are well validated, it is unclear whether these scoring systems can also work in non-ICU ward patients and thus provide clinicians with an alternative to the track-and-trigger systems.

In addition, the majority of currently available scoring systems quantify risk on the basis of one set of physiologic variables, usually at either hospital or ICU admission. Therefore, they do not take into account the many changes in a patient’s clinical status over the course of hospitalization. Prior studies have shown that sequential measurements of the Sequential Organ Failure Assessment (SOFA) score in the ICU are helpful and that trends in SOFA score over time can estimate prognosis independent of initial score on admission [28,29]. However, it is unknown whether sequential measurements of other scoring systems can provide additional prognostic information among non-ICU ward patients.

In a nested retrospective case-control study of non-ICU patients on the general hospital wards, we examined and compared the ability of nine prediction scores to estimate risk of clinical deterioration. In addition, we computed each score sequentially over the course of 72 hours in order to analyze their respective performance over time and to determine whether measuring changes in score improves discriminatory power.

## Materials and methods

### Description of cohort

The retrospective cohort included all adult patients who were admitted from the ER to the Jack D. Weiler Hospital (Bronx, NY, USA) or the Moses Division of the Montefiore Medical Center (Bronx, NY, USA) between 1 December 2009 and 31 March 2010, with a diagnosis of infection present on hospital admission, as defined by a validated list of International Classification of Diseases, Ninth Revision (ICD-9) codes indicative of infection [30,31] (Additional file 1). Only patients with an infection ICD-9 code present on admission were included in the cohort. Patients admitted directly to the ICU from the ER were excluded. All patients with limits on life-sustaining interventions were excluded. Both hospitals have a critical care consult service/RRT that responds to all requests for acute evaluation of clinical deterioration or ICU transfer. Any clinician or nurse is empowered and urged to call the RRT at the first sign of patient decline, defined as respiratory distress, threatened airway, respiratory rate of less than 8 or of more than 36, new hypoxemia (oxygen saturation (SaO_2_) of less than 90%) while on oxygen, new hypotension (systolic blood pressure of less than 90 mm Hg), new heart rate of less than 40 or of more than 140 beats per minute, sudden fall in level of consciousness, sudden collapse, Glasgow Coma Scale score decrease of more than 2 points, new limb weakness or facial asymmetry, repeated or prolonged seizures, or serious worry about a patient who did not qualify from any of the prior criteria.

### Definition of cases and controls

Cases are any patient with clinical deterioration, defined as ICU transfer, critical care consult for ICU transfer, rapid response evaluation, or in-hospital mortality. The index time for each case is defined as the earliest of these events.

One control was selected for each case by risk-set sampling and matched by hospital admission date. All controls survived to hospital discharge without an ICU admission, rapid response, or critical care consult. For each control, the index time was set so that the time from hospital admission to index time was equivalent for both the case and corresponding control. Finally, all controls must also have length of stay greater than the time to clinical deterioration of its corresponding case. This ensures that matched cases and controls had comparable hospital length of stay prior to clinical deterioration, allowing valid comparisons between these two groups within that time interval.

### Data collection

All clinical variables were collected retrospectively from either an electronic database or paper medical records. An electronic medical database query tool called *Clinical Looking Glass* was used to retrieve data such as patient demographics, vital signs, laboratory data, hospital admission and discharge dates, ICU transfer date, and ICD-9 codes. Paper medical records were reviewed for all data not found in the electronic medical records. Timing and activation of rapid response and critical care consults were obtained from a database maintained prospectively by the critical care team.

Baseline characteristics were available for all cases and controls except for comorbidities and source of infection, which were unavailable for 9% and 2% of the patients, respectively. Vital signs and laboratory measurements up to 72 hours before index time were collected for both cases and controls. For patients with hospital length of stay less than 72 hours (62% of patients), scores were calculated only for the time the patient was in the hospital. Among patients in the hospital, vitals and metabolic panel laboratory data over time were available for over 96% and 93% of patients, respectively. Arterial blood gases were performed in 30% of patients. This study was approved by the Einstein Montefiore Medical Center Institutional Review Board. In addition, the need for informed consent was waived by the Einstein Montefiore Medical Center Institutional Review Board because this was a retrospective study and no interventions were implemented.

### Prediction scores

We searched the literature for scores that were validated for use in the ER, ICU, or non-ICU medical wards. Scores were selected for this study if the scoring system (a) modeled risk for clinical deterioration such as ICU admission or death, (b) was validated with an acceptable area under the curve (AUC) of greater than 0.70 in a separate and independent cohort, and (c) consists of physiologic components which could be readily collected for ward patients. On this basis, nine prediction scores were selected: SOFA score, Predisposition/Infection/Response/Organ Dysfunction Score (PIRO), VitalPac Early Warning Score (ViEWS), Simple Clinical Score (SCS), Mortality in Emergency Department Sepsis (MEDS), Modified Early Warning Score (MEWS), Simplified Acute Physiology Score II (SAPS II), Acute Physiology and Chronic Health Evaluation Score II (APACHE II), and Rapid Emergency Medicine Score (REMS). APACHE II was selected in lieu of APACHE III because not all variables required for APACHE III were available in our floor patients. Characteristics of the nine prediction scores are shown in Additional file 2.

Scores were calculated for all patients at four time intervals: 0 to 12, 12 to 24, 24 to 48, and 48 to 72 hours before index time. If multiple laboratory values were available in a given time interval, the worst single value was used. If a laboratory value was missing, the value from the preceding time interval was used, if available. If there was also no available value from a preceding time interval, the laboratory value was assumed to be normal, similar to prior studies [32]. Glasgow Coma Scale is not routinely measured on non-traumatic patients at our hospital, similar to prior studies [13]. Instead, the alert/verbal/painful/unresponsive level was used and converted to a near-equivalent Glasgow Coma Scale value [33].

### Statistical analysis

Univariate analysis was performed by using two-tailed Fisher exact test, Student *t* test, or Wilcoxon rank-sum test as appropriate. To evaluate discriminatory power, area under the receiver operating characteristic curve (AUC) was calculated for each score. To compare two AUC measurements with equal sample size, DeLong’s non-parametric approach was used. To compare two AUC measurements with unequal sample size, we used an *ad hoc* z-test assuming that the correlation between them was 0.5. In keeping with Hosmer and Lemeshow [34], an AUC of at least 0.70 was defined as “acceptable discrimination” and an AUC of at least 0.80 was defined as “excellent discrimination”.

To evaluate changes in score over time, a mixed-effects linear model was applied after adjusting for baseline differences between cases and controls, such as age, gender, pneumonia, congestive heart failure, and severe sepsis. To examine between-group and between-time differences, we constructed and tested pertinent contrasts in the model. We selected the best fitting model across different dependent score variables that yielded the smallest Akaike information criterion (AIC) value, since the set of the independent variables was identical for all dependent variables in all models.

For each scoring model, optimal score cutoffs were determined by using receiver operating characteristic (ROC) analysis. Thresholds were selected on the basis of the models with the highest Youden index. To construct the clinical prediction rule that incorporated trends in score over time, the combination of earliest score threshold and change in score thresholds which produced the highest Youden index was selected. A multivariate regression model was employed to estimate the odds ratio after adjusting for baseline differences between cases and control. A *P* value of not more than 0.05 was considered statistically significant. All statistical analyses were performed by using Statistical Analysis System 9.3 (SAS Institute Inc., Cary, NC, USA).

## Results

In our cohort of 5,188 patients admitted with infection, we evaluated 328 cases and 328 matched controls. Of the 328 patients with clinical deterioration, 142 were admitted to the ICU, 200 received a rapid response or consult for ICU admission, and 110 died during hospitalization. By definition, all 328 cases experienced an ICU admission, consult for ICU admission, rapid response, or death during hospitalization or a combination of these. The median time from hospital admission to clinical deterioration was 32 hours (interquartile range of 12 to 124 hours).

Baseline characteristics of the cohort are presented in Table 1. Compared with controls, cases were generally older, more likely to be male, and more likely to be admitted from the nursing home. In addition, cases had more severe sepsis and pneumonia as the source of infection.

**Table 1:** Baseline characteristics of cases and controls

**Characteristics**	**Controls**	**Cases**	***P *** **value**
	**(n = 328)**	**(n = 328)**	
Demographics			
**Age, median (IQR)**	**64 (49-78)**	**67 (55-79)**	**0.01**
Body mass index, median (IQR)	28 (23-32)	27 (23-31)	0.3
**Female, n (%)**	**207 (63)**	**173 (53)**	**0.01**
**Race, n (%)**			**0.02**
White, non-Hispanic	60 (18)	93 (28)	
Black, non-Hispanic	111 (34)	96 (29)	
Hispanic	134 (41)	109 (33)	
Other	23 (7)	30 (9)	
Service, n (%)			0.2
Medical	269 (82)	255 (78)	
Surgical	59 (18)	73 (22)	
Suspected source of infection, n (%)			
**Pneumonia**	**84 (26)**	**135 (42)**	**<0.001**
Urinary tract	92 (28)	73 (22)	0.09
Skin or soft tissue	20 (6)	14 (4)	0.3
Peritonitis	3 (1)	9 (3)	0.14
Other	134 (41)	119 (36)	0.3
Comorbidities, n (%)			
**Severe sepsis**	**106 (32)**	**248 (76)**	**<0.001**
Chronic liver disease	77 (23)	83 (25)	0.6
Chronic pulmonary disease	136 (41)	123 (38)	0.3
Chronic renal disease	82 (25)	89 (27)	0.6
**Congestive heart failure**	**97 (30)**	**125 (38)**	**0.02**
Diabetes mellitus	122 (41)	127 (43)	0.7
History of myocardial infarction	49 (15)	57 (17)	0.5
Human immunodeficiency virus	21 (6)	15 (5)	0.4
Malignancy	61 (19)	76 (23)	0.17
Metastatic	23 (7)	29 (9)	0.5

*IQR,* interquartile range.

### Comparison of score discrimination at different time intervals

To evaluate discriminatory power, AUC was computed for all time intervals, as displayed in Table 2. At the 0- to 12-hour interval, all scores except REMS performed with acceptable discrimination (AUC ≥0.70) and had roughly equivalent AUC. Although SOFA performed the best with an AUC of 0.78 (95% confidence interval (CI) 0.74 to 0.81), this was not significantly higher than PIRO (AUC 0.76, *P* = 0.36), ViEWS (AUC 0.75, *P* = 0.28), SCS (0.74, *P* = 0.15), MEDS (0.74, *P* = 0.09), or MEWS (0.73, *P* = 0.07). However, at the 12- to 72-hour intervals, all scores, with the exception of MEDS, no longer performed with acceptable discrimination (AUC <0.70).

**Table 2:** Comparison of areas under the receiver operating curves for the nine scoring systems

**Score**	**0-12 hours**	**12-24 hours**	**24-48 hours**	**48-72 hours**
SOFA	**0.78**^**a **^**(0.74-0.81)**	**0.68**^**a **^**(0.63-0.73)**	0.66 (0.60-0.71)	0.64 (0.57-0.71)
PIRO	0.76 (0.72-0.79)	0.66 (0.61-0.71)	0.66 (0.61-0.72)	0.68 (0.61-0.75)
ViEWS	0.75 (0.71-0.79)	0.67 (0.62-0.72)	0.64 (0.58-0.69)	0.66 (0.59-0.73)
SCS	0.74 (0.70-0.78)	0.67 (0.62-0.72)	0.63 (0.57-0.69)	0.63 (0.56-0.71)
MEDS^b^	0.74 (0.70-0.78)	0.68 (0.63-0.73)	**0.69**^**a **^**(0.63-0.74)**	**0.71**^**a **^**(0.64-0.78)**
MEWS	0.73 (0.69-0.77)	0.66 (0.61-0.71)	0.59 (0.53-0.65)	0.60 (0.52-0.67)
SAPS II	0.73 (0.69-0.77)	0.67 (0.61-0.72)	0.61 (0.55-0.67)	0.60 (0.53-0.68)
APACHE II	0.72 (0.68-0.76)	0.66 (0.61-0.71)	0.61 (0.55-0.67)	0.60 (0.52-0.67)
REMS	0.67 (0.62-0.71)	0.63 (0.57-0.68)	0.55 (0.49-0.61)	0.59 (0.52-0.66)

Areas under the receiver operating characteristic curves along with 95% confidence intervals are displayed. ^a^Denotes best performing score at each time interval. ^b^Scores where AUC at 0 to 12 hours is NOT significantly higher than AUC at 12 to 24 hours, 24 to 48 hours, and 48 to 72 hours. *APACHE II,* Acute Physiology and Chronic Health Evaluation Score II; *MEDS,* Mortality in Emergency Department Sepsis; *MEWS,* Modified Early Warning Score; *PIRO,* Predisposition/Infection/Response/Organ Dysfunction Score; *REMS,* Rapid Emergency Medicine Score; *SAPS II,* Simplified Acute Physiology Score II; *SCS,* Simple Clinical Score; *SOFA,* Sequential Organ Failure Assessment; *ViEWS,* VitalPac Early Warning Score.

### Change in scores over time

**Figure 1:**
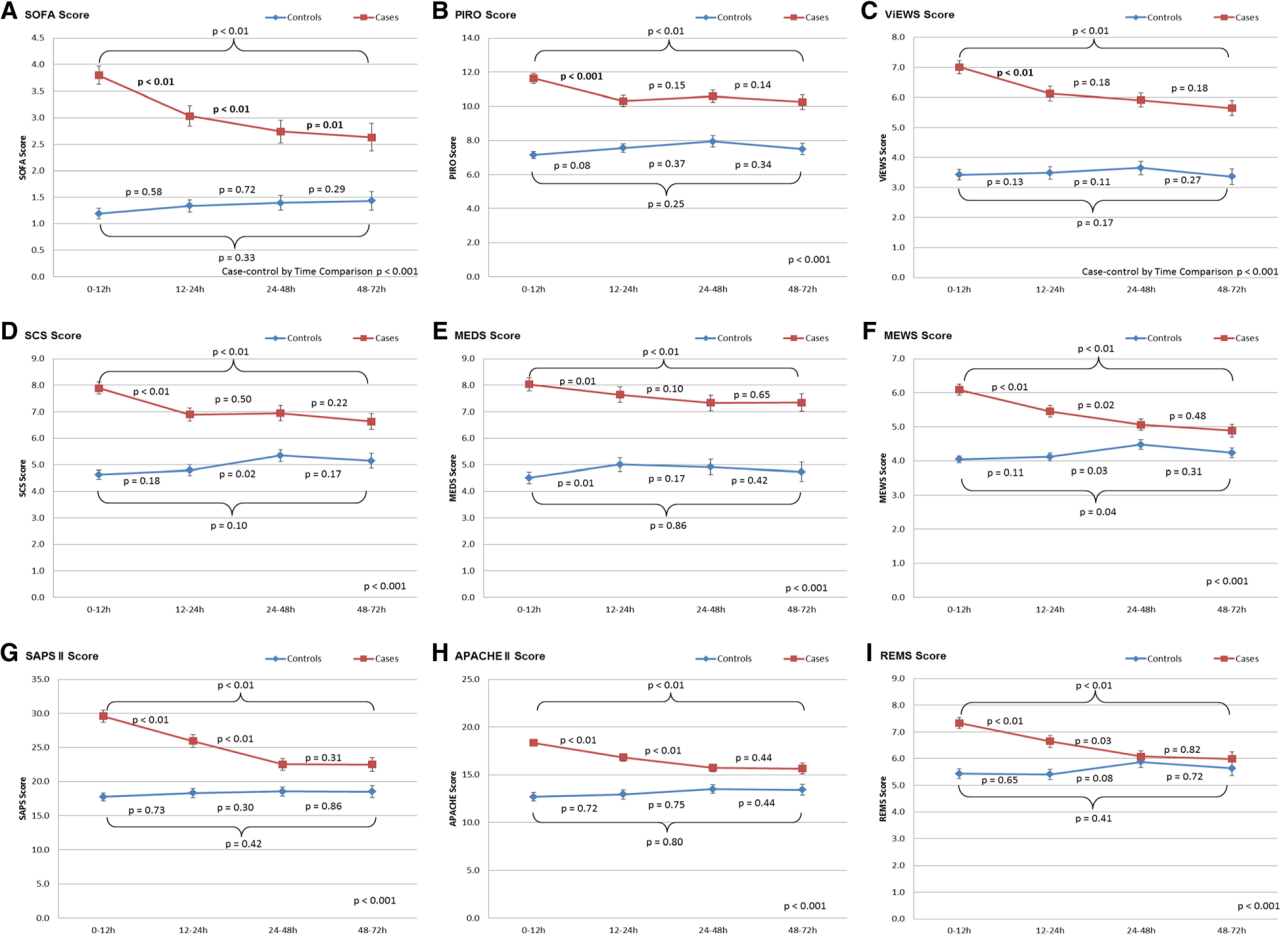
**Plot of average scores for cases and controls with respect to time.***P* values reflect pair-wise comparisons between consecutive time intervals, after adjusting for age, gender, severe sepsis, pneumonia, and congestive heart failure.

To characterize how scores changed relative to time of clinical deterioration, plots of average scores for case and controls are shown in Figure 1. For each model, average scores of cases were higher than average scores of controls at every time interval (*P* <0.01).

For all models, average scores of cases increased closer to time of clinical deterioration (*P* <0.05). For the MEWS, SAPS II, APACHE II, and REMS scoring models, this increase can be detected as early as 12 to 24 hours before deterioration (*P* <0.05). For SOFA, this increase can be detected even earlier at 24 to 48 hours before clinical deterioration. That is, the average SOFA score of cases during the 24- to 48-hour interval was significantly higher than during the 48- to 72-hour interval (*P* = 0.01). In contrast, average scores of controls did not increase closer to the index time.

### Exploratory analysis for models that incorporate change in score

To evaluate whether sequential measurements of a scoring system yielded additional prognostic value, we created and examined a clinical decision rule that used change in score (Δ-score), and compared it with traditional models that use only one set of measurements on admission. We applied this comparison to SOFA because it was the best performing score over time in our analysis.

First, we assessed a model that used the earliest available SOFA score. However, this model performed poorly, with a sensitivity of 76% and a specificity of 50% (Table 3). Next, we evaluated a model that used the highest SOFA score within 72 hours. This model performed better, with a good sensitivity of 74% but a poor specificity of 66%.

**Table 3:** Performance of three Sequential Organ Failure Assessment models

**Model**	**Sensitivity**	**Specificity**	**OR (95% CI)**	**OR**_**adj **_**(95% CI)**^**a**^
Earliest SOFA ≥1	76%	50%	3.23 (2.28-4.58)	2.26 (1.36-3.78)
Peak SOFA ≥2	74%	66%	5.63 (3.95-8.00)	3.34 (2.26-5.24)
Earliest SOFA ≥3 or ΔSOFA >0	75%	72%	7.85 (5.14-12.00)	5.89 (3.62-9.57)

Earliest Sequential Organ Failure Assessment (SOFA) represents the earliest available SOFA score within 72 hours of clinical deterioration. Peak SOFA represent highest SOFA score within 72 hours of clinical deterioration. ΔSOFA represent changes in consecutive SOFA scores. Thresholds selected based on receiver operating characteristic analysis. ^a^Adjusted odds ratio derived after adjusting for age, gender, severe sepsis, pneumonia, and congestive heart failure. CI, confidence interval; OR, odds ratio; OR_adj_, adjusted odds ratio.

**Figure 2:**
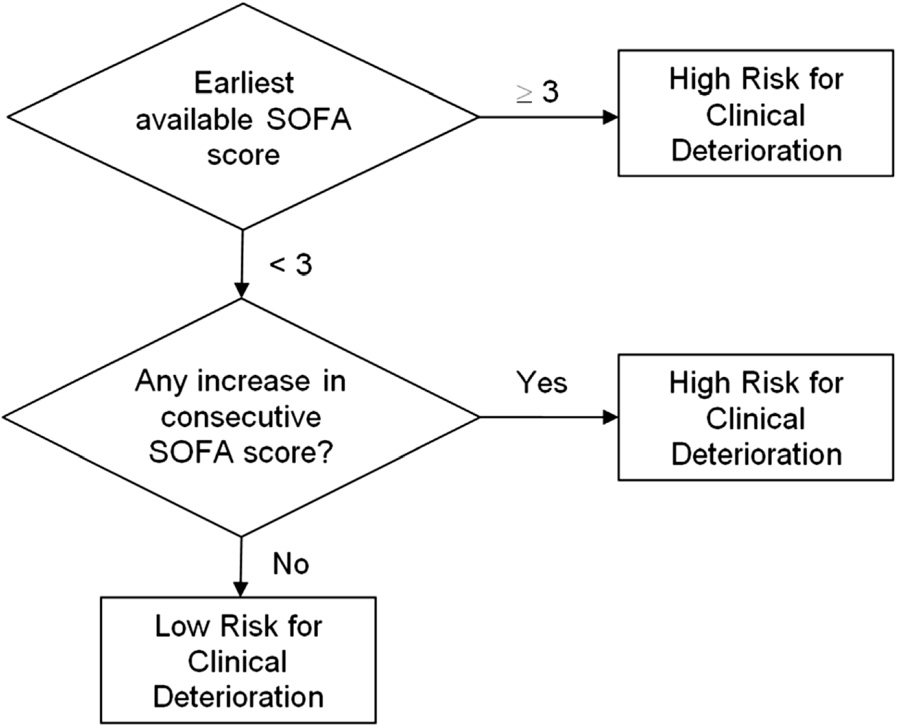
Clinical decision rule which incorporate both earliest available Sequential Organ Failure Assessment (SOFA) score and changes in SOFA score.

Finally, we constructed the clinical decision rule described in Figure 2. This model uses both the earliest available SOFA score and Δ-score and was found to perform even better, with a sensitivity of 75% and a specificity of 72%. Even after baseline differences between cases and controls (like age, gender, severe sepsis, pneumonia, and congestive heart failure) were adjusted for, patients who met the clinical decision rule criteria are almost six times more likely to clinically deteriorate compared with patients who did not (adjusted odds ratio (OR_adj_) 5.89, 95% CI 3.62 to 9.57) (Table 3).

### Mortality analysis

We performed a subgroup analysis, using mortality as the endpoint instead of clinical deterioration. At the 0- to 12-hour interval, seven of the eight scores performed similarly and had an AUC of greater than 0.80 (SOFA AUC 0.83, ViEWS 0.81, PIRO 0.87, SCS 0.83, MEDS 0.85, MEWS 0.82, SAPS II 0.83, and APACHE II 0.80) (Additional file 3). However, at the 12- to 72-hour intervals, only MEDS continued to predict for mortality with excellent discrimination (AUC >0.80).

In this subgroup analysis, the clinical decision rule described in Figure 2 performed even better, with a sensitivity of 79% and a specificity of 72% when predicting for mortality. Even after baseline differences between cases and controls were adjusted for, patients who met the clinical decision rule criteria are much more likely to die during hospitalization compared with patients who did not (OR_adj_ 13.3, 95% CI 5.3 to 33.3).

## Discussion

In a retrospective case-control study, we compared the discriminatory power of nine risk prediction scores and found that eight of the nine scores performed with similar and acceptable discrimination (AUC >0.70) within 12 hours prior to clinical deterioration. By measuring scores over time, we found that the some scores begin to worsen as early as 12 to 48 hours before time of clinical deterioration. Finally, we found that clinical decision rules that take advantage of the change in SOFA score over time have increased prognostic value over models that use a single SOFA measurement.

Our study demonstrates that both ER and ICU scoring systems can be used on non-ICU ward patients with similar performance compared with well-validated track-and-trigger systems such as MEWS and ViEWS. This gives clinicians on the floor a potential alternative to existing track-and-trigger systems. Interestingly, despite vast differences in the physiologic parameters used, all of these scores performed with very similar performance for ward patients.

Of note, the AUCs derived in our study are lower than those found in other studies [13,15-18]. However, the AUCs found in our study may be lower because many of these scores were originally derived and validated in a cohort of ICU or ER patients. Furthermore, these scores were all originally derived to calculate risk of mortality rather than clinical deterioration. Indeed, our subgroup analysis showed that when examining only mortality, the AUCs generated were higher and more closely resemble those from prior studies. However, we selected clinical deterioration as our endpoint because we wanted a score that could estimate need for critical care intervention and therefore prevent morbidity, not just mortality. For the purposes of our study, the endpoint of clinical deterioration was simply more relevant than using mortality, since not every patient who deteriorated to the point of requiring ICU evaluation necessarily died later on. By being able to anticipate clinical deterioration, clinicians can better administer early intervention measures or trigger RRT.

We found that scores performed better closer to time of clinical deterioration, which makes sense since physiologic parameters associated with certain events, such as cardiac arrest, may not be present long before the event. We discovered that MEDS performed with acceptable performance even 48 to 72 hours before time of deterioration. This finding may be explained by the fact that MEDS contains many baseline variables which do not change throughout hospitalization.

In studying the scores over time, we found that physiologic changes prior to clinical deterioration may be detected hours before the event. For patients who ultimately deteriorated, the average scores increased 12 to 24 hours prior to time of deterioration for the MEWS, SAPS II, APACHE II, and REMS scoring models. For the SOFA model, average scores of cases increased as early as 24 to 48 hours before time of deterioration. This early warning is significant and can provide clinicians with sufficient time to reassess patients. This also allows time for early interventional measures to take effect and improve outcomes. In the context of previous findings, one trial studying the impact of implementing a track-and-trigger system found that about 50% of deteriorating patients were not detected by the trigger system until less than 15 minutes prior to hospital death, cardiac arrest, or ICU admission [35]. Furthermore, since 76% of our cases had severe sepsis, serial assessment of these prediction scores may also help clinicians identify patients with worsening severe sepsis who may benefit from early goal-directed therapy.

For our exploratory analysis, we constructed a clinical decision rule that incorporated both changes in SOFA score and the earliest available SOFA score. Whereas the earliest score represents baseline severity of illness, the change in SOFA score reflects events during course of the hospitalization, such as clinical deterioration or inadequate response to therapeutic interventions. Thus, this rule captures both patients who were very sick on presentation or worsened during hospitalization. This clinical prediction rule was derived objectively from ROC analysis and is the first to use SOFA score in a cohort of non-ICU patients. More importantly, this decision rule has good discriminatory ability. Even after baseline differences were adjusted for, patients who meet the clinical decision rule criteria are almost six times more likely to deteriorate clinically compared with patients who did not.

However, with a sensitivity of 75%, many patients who would have deteriorated are still missed. However, it is still higher than the sensitivities of existing track-and-trigger systems (25% to 69%) [24]. Furthermore, its specificity of 72%, though comparable to that of track-and-trigger systems, means that many patients who satisfy the clinical decision rule will not deteriorate. This makes ICU admission for every at-risk patient impractical since stable patients will end up using scarce ICU resources. However, early identification of at-risk patients may still be useful. High-risk patients can receive closer follow-up, early intervention measures that can be done on the non-ICU wards, and benefit from earlier discussions on goals of care.

This study has a number of strengths. To the best of our knowledge, this was the first study to provide a head-to-head comparison of multiple ER and ICU prediction scores in a cohort of non-ICU ward patients. We assessed scores sequentially over time and, by using risk-set sampling and controlling for time to exposure, were able to account for time-dependent variables. Finally, we measured scores in the 72 hours before clinical deterioration. This study design allowed us to demonstrate the temporal trends of scores relative to time of deterioration rather than time of ICU admission.

However, we acknowledge some limitations. This was a retrospective case-control study involving chart reviews. Though not a prospective cohort study, this was a nested case-control study which has been shown to produce accurate sensitivities and specificities since both cases and controls are selected from a cohort of patients with similar backgrounds [36]. Nevertheless, the results and findings of this study need to be validated in a future prospective study. Our cohort was limited to patients with infection, although this was done in similar prior studies [2,5,13] since patients with infection constitute a substantial percentage of ward patients. Missing variables were assumed to be normal, although this was similar to prior studies [32]. Some variables, such as functional status variables and Glasgow Coma Scale, were not collected. However, these variables are not routinely assessed and documented on ward patients and therefore this omission may be in line with actual clinical practice. Instead, we converted alert/verbal/painful/unresponsive scale measurements into equivalent Glasgow Coma Scale values by using a previously verified conversion [33]. Finally, we did not have continuous monitoring of vital signs, the presence of which could have provided greater resolution of data.

## Conclusions

Both ER and ICU prediction scores can be used to estimate a ward patient’s risk of clinical deterioration, with good discriminatory ability comparable to that of existing track-and-trigger systems. We also found that some scores, such as SOFA, began to increase as early as 12 to 48 hours before time of clinical deterioration. Accordingly, we constructed a clinical decision rule for SOFA that used both the change in SOFA and baseline SOFA, and found that it performed well.

## Key messages

• ER and ICU risk prediction scores can prognosticate with good discrimination the risk of clinical deterioration in ward patients.

• By measuring some risk prediction scores (SOFA, MEWS, SAPS II, APACHE II, and REMS) over time, we find that the scores of cases begin to increase as early as 12 to 48 hours prior to time of clinical deterioration.

• Clinical decision rules that take advantage of the change in SOFA over time have increased prognostic value over models that use a single SOFA measurement.

## Abbreviations

APACHE: Acute Physiology and Chronic Health Evaluation; AUC: area under the curve; CI: confidence interval; ER: emergency room; ICD-9: International Classification of Diseases, Ninth Revision; ICU: intensive care unit; MEDS: Mortality in Emergency Department Sepsis; MEWS: Modified Early Warning Score; OR_adj_: adjusted odds ratio; PIRO: Predisposition/Infection/Response/Organ Dysfunction; REMS: Rapid Emergency Medicine Score; ROC: receiver operating characteristic; RRT: rapid response team; SAPS II: Simplified Acute Physiology Score II; SCS: simple clinical score; SOFA: Sequential Organ Failure Assessment; ViEWS: VitalPac Early Warning Score.

## Competing interests

The authors declare that they have no competing interests.

## Authors’ contributions

SY participated in study design, data collection, data management, and statistical analysis and drafted the initial manuscript. SL participated in study design and data collection. MH participated in statistical analysis and in the drafting and editing of the manuscript. GJS, RTS, and SG participated in data collection, interpretation of results, and review of the manuscript. MNG conceived the study, directed the study design and analysis, and revised all drafts of the manuscript. All authors read and approved the final manuscript.

## Supplementary Material

Additional file 1 International Classification of Diseases, Ninth Revision (ICD-9) codes used to assess diagnosis of infection. Click here for file

Additional file 2 Characteristics and components of the nine examined risk prediction scoring systems. Click here for file

Additional file 3 Area under the curve measurements for the nine scoring systems when using mortality as the endpoint. Click here for file
