# Supplementary material for: Comparison of risk prediction scoring systems for ward patients: a retrospective nested case-control study
Source: Crit Care. 2014 Jun 26;18(3):R132. doi: 10.1186/cc13947 (PMC4227284; doi:10.1186/cc13947)
Supplement: Additional file 1 — International Classification of Diseases, Ninth Revision (ICD-9) codes used to assess diagnosis of infection. [file cc13947-S1.docx]

Validated list of ICD-9-CM Codes used to identify infection.

001, Cholera; 002, Typhoid/paratyphoid fever; 003, Other salmonella infection; 004, Shigellosis; 005, Other food poisoning; 008, Intestinal infection not otherwise classified; 009, Ill-defined intestinal infection; 010, Primary tuberculosis infection; 011, Pulmonary tuberculosis; 012, Other respiratory tuberculosis; 013, Central nervous system tuberculosis; 014, Intestinal tuberculosis; 015, Tuberculosis of bone and joint; 016, Genitourinary tuberculosis; 017, Tuberculosis not otherwise classified; 018, Miliary tuberculosis; 020, Plague; 021, Tularemia; 022, Anthrax; 023, Brucellosis; 024, landers; 025, Melioidosis; 026, Rat-bite fever; 027, Other bacterial zoonoses; 030, Leprosy; 031, Other mycobacterial disease; 032, Diphtheria; 033, Whooping cough; 034, Streptococcal throat/scarlet fever; 035, Erysipelas; 036, Meningococcal infection; 037, Tetanus; 038, Septicemia; 039, Actinomycotic infections; 040, Other bacterial diseases; 041, Bacterial infection in other diseases not otherwise specified; 090, Congenital syphilis; 091, Early symptomatic syphilis; 092, Early syphilis latent; 093, Cardiovascular syphilis; 094, Neurosyphilis; 095, Other late symptomatic syphilis; 096, Late syphilis latent; 097, Other and unspecified syphilis; 098, Gonococcal infections; 100, Leptospirosis; 101, Vincent’s angina; 102, Yaws; 103, Pinta; 104, Other spirochetal infection; 110, Dermatophytosis; 111, Dermatomycosis not otherwise classified or specified; 112, Candidiasis; 114, Coccidioidomycosis; 115, Histoplasmosis; 116, Blastomycotic infection; 117, Other mycoses; 118, Opportunistic mycoses; 320, Bacterial meningitis; 322, Meningitis, unspecified; 324, Central nervous system abscess; 325, Phlebitis of intracranial sinus; 420, Acute pericarditis; 421, Acute or subacute endocarditis; 451, Thrombophlebitis; 461, Acute sinusitis; 462, Acute pharyngitis; 463, Acute tonsillitis; 464, Acute laryngitis/tracheitis; 465, Acute upper respiratory infection of multiple sites/not otherwise specified; 481, Pneumococcal pneumonia; 482, Other bacterial pneumonia; 485, bronchopneumonia with organism not otherwise specified; 486, Pneumonia, organism not otherwise specified; 491.21, Acute exacerbation of obstructive chronic bronchitis; 494, Bronchiectasis; 510, Empyema; 513, Lung/mediastinum abscess; 540, Acute appendicitis; 541, Appendicitis not otherwise specified; 542, Other appendicitis; 562.01, Diverticulitis of small intestine without hemorrhage; 562.03, Diverticulitis of small intestine with hemorrhage; 562.11, Diverticulitis of colon without hemorrhage; 562.13, Diverticulitis of colon with hemorrhage; 566, Anal and rectal abscess; 567, Peritonitis; 569.5, Intestinal abscess; 569.83, Perforation of intestine; 572.0, Abscess of liver; 572.1, Portal pyemia; 575.0, Acute cholecystitis; 590, Kidney infection; 597, Urethritis/urethral syndrome; 599.0, Urinary tract infection not otherwise specified; 601, Prostatic inflammation; 614, Female pelvic inflammation disease; 615, Uterine inflammatory disease; 616, Other female genital inflammation; 681, Cellulitis, finger/toe; 682, Other cellulitis or abscess; 683, Acute lymphadenitis; 686, Other local skin infection; 711.0, Pyogenic arthritis; 730, steomyelitis; 790.7, Bacteremia; 996.6, Infection or inflammation of device/graft; 998.5, Postoperative infection; 999.3, Infectious complication of medical care not otherwise classified; 995.91, sepsis; 995.92, severe sepsis; 785.52, septic shock.
