# Supplementary material for: Comparison of risk prediction scoring systems for ward patients: a retrospective nested case-control study
Source: Crit Care. 2014 Jun 26;18(3):R132. doi: 10.1186/cc13947 (PMC4227284; doi:10.1186/cc13947)
Supplement: Additional file 2 — Characteristics and components of the nine examined risk prediction scoring systems. [file cc13947-S2.docx]

**Supplemental Table 1** Characteristics of the nine scoring models

|  |  |  |  |  |  |
| --- | --- | --- | --- | --- | --- |
| Scoring Model [Citation] | Population | Predicted Outcome | Original Derivation Cohort | Original Validation Cohort | No. Hospitals |
| SOFA [1]^a^ | ICU | Mortality | - | 1449 patients | 40 |
| PIRO [2] | ED w/ infxn | Mortality | 2132 patients | 5622 patients | 2 |
| ViEWS [3] | MAU | Mortality | 35585 patients | 35585 patients | 1 |
| SCS [4] | AMU | Mortality | 6736 patients | 3228 patients | 1 |
| MEDS [5] | ED w/ infxn | Mortality | 2070 patients | 1109 patients | 1 |
| MEWS [6]^a^ | MAU | ICU Adm, Mortality | - | 709 patients | 1 |
| SAPS II [7] | ICU | Mortality | 8549 patients | 4603 patients | 137 |
| APACHE II [8] | ICU | Mortality | - | 5815 patients | 13 |
| REMS [9] | ED | Mortality | 6003 patients | 6003 patients | 1 |

Citations refer to original validation study. *AMU* acute medical unit, *MAU* medical admissions unit, *SOFA* Sequential Organ Failure Assessment Score, *PIRO* Predisposition/Infection/Response/Organ Dysfunction Score, *ViEWS* VitalPac Early Warning Score, *SCS* Simple Clinical Score, *MEDS* Mortality in Emergency Department Sepsis, *MEWS* Modified Early Warning Score, *SAPS II* Simplified Acute Physiology Score II, *APACHE II* Acute Physiology and Chronic Health Evaluation Score II, *REMS* Rapid Emergency Medicine Score, w/ infxn with infection.

^a^ Scores were derived from expert opinion and therefore had no derivation cohort.

**Supplemental Table 2** Components of the nine scoring systems.

| Components | SOFA | ViEWS | PIRO | SCS | MEDS | MEWS | SAPS II | APACHE II | REMS |
| --- | --- | --- | --- | --- | --- | --- | --- | --- | --- |
| Baseline Demographics |  |  |  |  |  |  |  |  |  |
| Age |  |  | Y | Y | Y | Y | Y | Y | Y |
| Type of Admission |  |  |  |  |  |  | Y | Y |  |
| Metastatic Malignancy |  |  | Y |  | Y |  | Y |  |  |
| Nursing Home Resident |  |  | Y | Y | Y |  |  |  |  |
| Pneumonia |  |  | Y |  | Y |  |  |  |  |
| Vitals |  |  |  |  |  |  |  |  |  |
| Respiratory Rate |  | Y | Y | Y | Y | Y |  | Y | Y |
| Temperature |  | Y |  | Y |  | Y | Y | Y | Y |
| Heart Rate |  | Y | Y | Y |  | Y | Y | Y | Y |
| Blood pressure | MAP | SBP | SBP | SBP |  | SBP | SBP | MAP | MAP |
| Organ Dysfunction |  |  |  |  |  |  |  |  |  |
| SaO2 and/or PaO2 | PFR | SaO2 | SaO2 | SaO2 | SaO2 |  | PaO2 | PaO2 | SaO2 |
| Total Bilirubin | Y |  |  |  |  |  | Y |  |  |
| Mental Status | GCS | AVPU |  | AMS, Coma | AMS | AVPU | GCS | GCS | GCS |
| White Blood Cells or Bands | |  | Bands |  | Bands |  | WBC | WBC |  |
| Platelets | Y |  | Y |  | Y |  |  |  |  |
| Metabolic Panel |  |  |  |  |  |  |  |  |  |
| Sodium |  |  |  |  |  |  | Y | Y |  |
| Potassium |  |  |  |  |  |  | Y | Y |  |
| Bicarbonate or pH |  |  |  |  |  |  | Bicarb | pH |  |
| BUN or Creatinine | Cr |  | BUN |  |  |  | BUN | Cr |  |
| Other |  | Supple-mental O_2_ | Lactate, COPD, Chronic Liver Disease | Functional Status†, SOB, Abn EKG, New Stroke, Diabetes | Septic Shock |  | AIDS | Hematocrit, Glucose, Hx of severe organ insufficiency |  |

See Supplemental Table 1 legend for scoring system abbreviations and citations. MAP = mean arterial pressure; SBP = systolic blood pressure; PFR = PaO_2_/FiO_2_ ratio; PaO_2_ = partial pressure of oxygen; SaO_2_ = saturation of oxygen; GCS = Glasgow Coma Scale; AMS = altered mental status; AVPU = alert/verbal/pain/unresponsive scale; WBC = white blood cells; Bicarb = Bicarbonate; BUN = blood urea nitrogen, Cr = Creatinine, COPD = chronic pulmonary obstructive disease, SOB = shortness of breath, Abn = abnormal, Hx = History.

†Functional Status, including variables such as "unable to stand unaided" or "prior to current illness, spent some part of daytime in bed", were not collected because it was not commonly assessed and recorded in the chart.
