# Supplementary material for: Comparison of risk prediction scoring systems for ward patients: a retrospective nested case-control study
Source: Crit Care. 2014 Jun 26;18(3):R132. doi: 10.1186/cc13947 (PMC4227284; doi:10.1186/cc13947)
Supplement: Additional file 3 — Area under the curve measurements for the nine scoring systems when using mortality as the endpoint. [file cc13947-S3.docx]

**Supplemental Table 3** Comparison of areas under the receiver operating curves (AUC) for the nine scoring systems when using mortality as the endpoint.

| Score | 0-12 hours | 12-24 hours | 24-48 hours | 48-72 hours |
| --- | --- | --- | --- | --- |
| SOFA | 0.83 (0.77-0.88) | 0.76 (0.69-0.83) | 0.75 (0.67-0.83) | 0.74 (0.64-0.83) |
| PIRO | 0.87 (0.82-0.92) | 0.79 (0.73-0.86) | 0.76 (0.68-0.84) | 0.78 (0.69-0.86) |
| ViEWS | 0.81 (0.76-0.87) | 0.77 (0.70-0.84) | 0.72 (0.64-0.81) | 0.78 (0.70-0.87) |
| SCS | 0.83 (0.78-0.89) | 0.78 (0.71-0.85) | 0.74 (0.66-0.83) | 0.74 (0.65-0.84) |
| MEDS | 0.85 (0.79-0.90) | 0.81 (0.74-0.87) | 0.81 (0.74-0.89) | 0.82 (0.74-0.90) |
| MEWS | 0.82 (0.77-0.88) | 0.77 (0.70-0.84) | 0.70 (0.62-0.79) | 0.73 (0.64-0.83) |
| SAPS II | 0.83 (0.77-0.89) | 0.77 (0.70-0.84) | 0.74 (0.65-0.82) | 0.74 (0.65-0.84) |
| APACHE II | 0.80 (0.74-0.86) | 0.75 (0.67-0.82) | 0.73 (0.64-0.81) | 0.74 (0.64-0.83) |
| REMS | 0.75 (0.65-0.79) | 0.70 (0.63-0.78) | 0.63 (0.53-0.72) | 0.64 (0.54-0.75) |

Areas under the receiver operating characteristic curves along with 95% confidence intervals are displayed. Analysis was applied to the subgroup of cases that died during hospitalization (n = 110) along with their corresponding controls (n = 110). See Table 2 legend for expansion of abbreviations.
